# Hydrogen sulfide as a vasculoprotective factor

**DOI:** 10.1186/2045-9912-3-9

**Published:** 2013-04-29

**Authors:** Eloise Streeter, Hooi H Ng, Joanne L Hart

**Affiliations:** 1 School of Medical Sciences and Health Innovations Research Institute (HIRi), RMIT University, PO Box 70, Bundoora, Vic, 3083, Australia

**Keywords:** Hydrogen sulfide, Vasculoprotective, Atherosclerosis oxidative stress

## Abstract

Hydrogen sulfide is a novel mediator with the unique properties of a gasotransmitter and many and varied physiological effects. Included in these effects are a number of cardiovascular effects that are proving beneficial to vascular health. Specifically, H_2_S can elicit vasorelaxation, prevention of inflammation and leukocyte adhesion, anti-proliferative effects and anti-thrombotic effects. Additionally, H_2_S is a chemical reductant and nucleophile that is capable of inhibiting the production of reactive oxygen species, scavenging and neutralising reactive oxygen species and boosting the efficacy of endogenous anti-oxidant molecules. These result in resistance to oxidative stress, protection of vascular endothelial function and maintenance of blood flow and organ perfusion. H_2_S has been shown to be protective in hypertension, atherosclerosis and under conditions of vascular oxidative stress, and deficiency of endogenous H_2_S production is linked to cardiovascular disease states. Taken together, these effects suggest that H_2_S has a physiological role as a vasculoprotective factor and that exogenous H_2_S donors may be useful therapeutic agents. This review article will discuss the vascular effects and anti-oxidant properties of H_2_S as well as examine the protective role of H_2_S in some important vascular disease states.

## Introduction

Hydrogen sulfide is now a recognised gaseous mediator and induces many and varied biological effects [1]. Several cardiovascular actions of H_2_S have been described, including vasorelaxation, prevention of inflammation and leukocyte adhesion, anti-proliferative effects, anti-thrombotic effects, resistance to oxidative stress and protection against ischemia-reperfusion injury. These result in protection of endothelial function, resistance to vascular remodelling and maintenance of blood flow and organ perfusion. Taken together, these effects suggest that H_2_S has a physiological role as a vasculoprotective factor. This review examines the evidence that H_2_S is an important vascular regulator and protectant.

### H_2_S production, storage and metabolism

H_2_S is produced endogenously via the metabolism of cysteine and/or homocysteine [2], by the enzymes cystathionine-β-synthase (CBS, EC 4.2.1.22) [3] and cystathionine-γ-lyase (CSE, EC 4.4.1.1) [4]. 3-mercaptopyruvate sulfurtransferase (3-MST, EC 2.8.1.2) can also generate H_2_S acting in concert with cysteine aminotransferase (EC 2.6.1.75) to metabolise cysteine, generating pyruvate and H_2_S [5]. CBS is a major contributor to H_2_S production in the brain, whilst CSE levels predominate in most peripheral tissues. 3-MST appears to contribute to H_2_S production in both the periphery and central nervous system [5,6]. In the vascular system CSE is primarily expressed in vascular smooth muscle cells but there is also evidence that it is expressed in the endothelium [7,8].

H_2_S is metabolized by mitochondrial oxidative modification that converts sulfide into thiosulfate, which is converted further into sulfite and finally sulfate, which is the major end product of H_2_S metabolism [9]. H_2_S consumption in the presence of O_2_ is high [10], thus H_2_S production is offset by rapid clearance, resulting in low basal levels of H_2_S. In addition to high clearance H_2_S may also be stored as acid-labile sulphur [11] or bound sulfane sulphur within cells [12]. The metabolic turnover of H_2_S and concentrations of the gas generated *in vivo* during cell stimulation are yet to be fully elucidated and will be an area of importance in H_2_S biology future research.

### Gasotransmitter and chemical properties

Gaseous mediators or gasotransmitters are a relatively new class of signalling molecules, These gases share many features in their production and action but differ from classical signalling molecules. Advantages of gases as signalling molecules include their small size which allows easy access to a variety of target sites that would not be accessible by larger molecules. They easily cross membranes, are labile with short half-lives and are made on demand. They are not stored in their native form as they can’t be constrained by vesicles and need to be bound for storage or rely upon *de novo* synthesis. They can have endocrine, paracrine, autocrine or even intracrine effects. It is also interesting that all the molecules confirmed as gasotransmitters (nitric oxide (NO), carbon monoxide (CO), H_2_S) were all considered only as toxic molecules until their endogenous production and effects were determined.

About 80% of H_2_S molecules dissociate into hydrosulfide anion (HS^-^) at physiological pH 7.4 in plasma and extracellular fluids [13]. HS^-^ is a potent one-electron chemical reductant and nucleophile that is capable of scavenging free radicals by single electron or hydrogen atom transfer [14,15] Thus, H_2_S should readily scavenge reactive nitrogen species (RNS) and reactive oxygen species (ROS) [16]. It is also now established that H_2_S can signal via sulhydration of proteins [17], and much research is ongoing in this area.

### H_2_S effects on blood vessels

**Figure 1:**
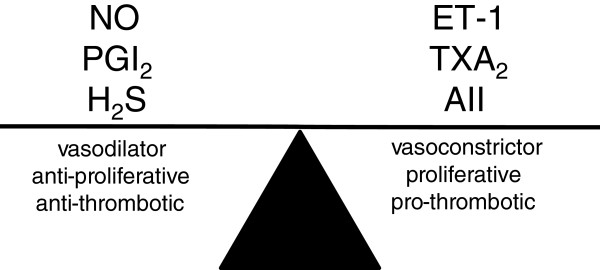
**The balance between vascular relaxant and constrictor factors.** The balance of vasoactive factors maintains vascular tone. Vasodilator factors also have anti-proliferative and anti-thrombotic effects, whereas vasoconstrictor factors tend to also have proliferative and/or pro-thrombotic effects. Increases in vasoconstrictor factors or decreases in vasorelaxant factors favour vascular contraction and other pathophysiological changes detrimental to vascular health [18]. PGI_2_: prostacyclin, ET-1: endothelin-1, TXA_2_: thromboxane A_2_, AII: angiotensin II.

Endothelium derived substances that cause vasodilatation (eg NO, prostacyclin) are anti-proliferative and anti-thrombotic while constrictor factors (endothelin-1, thromboxane A_2_) are proliferative and pro-coagulant. Thus the vasodilators can be considered vasculoprotective, as they protect and promote blood flow and a balance of endothelium-derived relaxing and contracting factors is required for a healthy vascular function [18]. H_2_S is produced in blood vessels by both endothelial cells and vascular smooth muscle has these same vasculoprotective properties (Figure 1). These are further discussed below.

### Vasorelaxation elicited by H_2_S

H_2_S induced vasorelaxation in peripheral vessels may be mediated by various mechanisms, including opening of potassium channels, blockade of voltage-gated Ca^2+^ channels, enhanced production or activity endothelial derived factors, such as NO, PGI_2_ and EDHF and decreased pH_i_. The vasorelaxant effect occurs in both large conduit [19-22] and small resistance-like blood vessels [7,23,24] and is physiologically relevant since an inhibition of CSE in isolated mouse aorta *in vitro* causes significant vascular contraction [19] and most importantly, mice deficient in CSE are hypertensive and have endothelial dysfunction [8].

### Platelet inhibition

Limited data is available on the action of H_2_S on platelets, although it has been reported that H_2_S can decrease platelet aggregation [25]. A recent *in vitro* study showed that platelet adhesion to collagen and fibrinogen, the first step in platelet activation and aggregation, was significantly reduced by nanomolar concentrations of NaHS. Additionally, platelet superoxide production was also inhibited although the mechanism of this effect was not examined [26]. Whilst platelet adhesion and aggregation are important for vascular haemostatis in trauma, they are undesirable under conditions of vascular inflammation and atherosclerosis, so further investigation into the role of H_2_S in platelet function is warranted.

### H_2_S as an anti-oxidant in the vasculature

Reactive oxygen species (ROS) can be divided into free radicals, such as superoxide (O_2_˙^-^) and hydroxyl (OH˙); non-radicals, such as hydrogen peroxide (H_2_O_2_); and reactive nitrogen species, such as NO (technically, NO˙, since it is a radical gas, with an unpaired electron) and peroxynitrite (ONOO^-^). In vascular cells, there are multiple sources for the generation of ROS, including mitochondria, cyclooxygenases and NADPH oxidases, xanthine oxidase, cyclo-oxygenase [27]. In mammalian tissues, reactive oxygen species (ROS) such as superoxide (O_2_^•-^) are produced under both pathological and physiological conditions. They are essential for the immunological defence mechanism of phagocytes, however, overproduction of ROS has detrimental effects on tissues including the vasculature. Excess ROS levels or oxidative stress are implicated in the pathology and progression of cardiovascular disease [28]. Excess levels of ROS can compromise the antioxidant defence mechanism of the cells and react with cellular macromolecules such as lipids, proteins, membrane bound polyunsaturated fatty acids and DNA leading to irreversible cellular damage [29]. Furthermore, perhaps the best characterized mechanism by which oxidative stress can cause dysfunction and damage to vascular cells is via the scavenging of vasoprotective nitric oxide by O_2_^•-^ leading to a reduction its biological half-life [30].

Superoxide is the parent ROS molecule in all cells. It can be generated in vascular cells by NADPH oxidases (or “Nox oxidases”), uncoupled endothelial NO synthase (eNOS), the mitochondrial enzyme complexes, cytochrome P450 and xanthine oxidase [27]. The Nox oxidases are the only enzymes discovered to date that have the primary function of generating superoxide (Nox1-3) and hydrogen peroxide (Nox4). This family of enzymes compromises two membrane-bound subunits, the Nox catalytic subunit and p22phox as well as various combinations of cytoplasmic subunits [31]. In the aorta at least 3 isoforms of Nox oxidase are expressed, Nox1-, Nox2- and Nox4-containing Nox oxidases. Importantly, ROS are generated at low levels in cerebral vessels and act there as signalling molecules involved in vascular regulation [32]. Excessive production of ROS, in particular superoxide (O_2_^•-^) from Nox oxidases is implicated as a key mediator of endothelial dysfunction (loss of NO bioavailability) associated with many cardiovascular diseases, including atherosclerosis to diabetic vascular disease and hypertension [33].

### H_2_S as a ROS scavenger

H_2_S is a potent one-electron chemical reductant and nucleophile that is theoretically capable of scavenging free radicals by single electron or hydrogen atom transfer [14]. Thus, H_2_S may participate in many reactions [34] and is reported to readily scavenge reactive oxygen and nitrogen species such as peroxynitrite [35], superoxide [36], hydrogen peroxide [37], hypochlorous acid [38] and lipid hydroperoxides [14]. However the kinetics, reactivity and mechanism of H_2_S/HS^-^ interactions with ROS are poorly understood under physiological conditions [14]. H_2_S has been reported to inhibit superoxide production in human endothelial cells [39] and vascular smooth muscle cells [40] by reducing Nox oxidase expression and activity. However it is not known if this activity is physiologically relevant, or whether H_2_S can protect against oxidative-stress driven vascular dysfunction. In addition, H_2_S is reported to increase glutathione levels and bolster endogenous anti-oxidant defences [41]. Collectively, these findings suggest that this molecule may be a useful vasoprotective agent.

### H_2_S as an inhibitor of ROS formation

H_2_S has also been shown to be important in regulating mitochondrial function [42] and can reduce mitochondrial ROS formation [43]. Hyperglycaemia induced overproduction of ROS was reversed with H_2_S treatment and furthermore, endogenously produced H_2_S acts to protect endothelial function from hyperglycaemic oxidative stress [44]. NaHS 30-50 μM protects rat aortic smooth muscle cells from homocysteine-induced cytotoxicity and reactive oxygen species generation, and furthermore NaHS-induced protective effects were synergistic with endogenous anti-oxidants [36]. This study suggests that H_2_S is capable of reducing production of H_2_O_2_, ONOO^-^ and O_2_^-^ in a time and concentration dependent manner. The mechanism of this effect was not established, however H_2_S at nanomolar concentrations has been reported to inhibit superoxide formation in human endothelial cells [39] and vascular smooth muscle cells [40] by reducing Nox oxidase expression and activity.

### H_2_S effects on endogenous anti-oxidants

NaHS has been shown to protect neurons from oxidative stress by boosting glutathione levels [41] and others have also shown that NaHS increases the activity of endogenous anti-oxidants such as superoxide dismutase, glutathione perioxidase and glutathione reductase [36,43,45,46]. There is now increasing evidence that H_2_S has a role in regulating the nuclear factor erthyroid 2 (NF-E2)-related factor 2 (Nrf2) pathway. Nrf2 is a key transcription regulator of inducible cell defence. In the presence of electrophiles and/or reactive oxygen species, Nrf2 accumulates, translocates to the cell nucleus and binds with antioxidant response elements (AREs). These are located within the promoter regions of an array of cell defence genes, regulating both basal and inducible expression of anti-oxidant proteins, detoxification enzymes and other stress response proteins [47].

Recent studies have shown that H_2_S donor treatment can induce Nrf2 expression [48,49] enhance Nrf2 translocation to the nucleus [50,51] and activate Nrf2 signalling [52], resulting in reduced oxidative stress and cardioprotection. The mechanism of the upregulation of Nrf2 by H_2_S is under investigation with recent reports that H_2_S inactivates the negative regulator of Nrf2, Keap1 [53,54] resulting in the Nrf2 mediated induction of cytoprotective genes.

**Figure 2:**
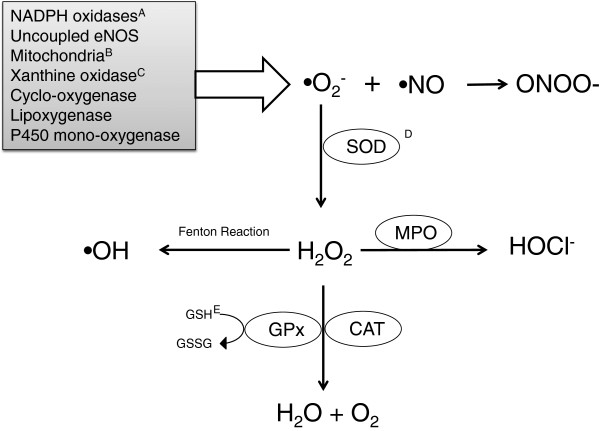
**Sources of vascular reactive oxygen species and potential protective effects of H**_**2**_**S.** Schema showing the major vascular sources of superoxide, the parent reactive oxygen species. H_2_S has been shown to inhibit **A**. NADPH oxidase activity and expression [39,40], **B**. mitochondrial ROS production [43], and possibly **C**. xanthine oxidase activity [74]. Additionally, H_2_S has been reported to scavenge ROS [35-38] and also promote the actions of **D**. SOD [43] and **E**. GSH [41]. SOD: superoxide dismutase, MPO: myeloperoxidase, CAT: catalase, GPx: glutathione peroxidase, GSH: reduced glutathione, GSSG, oxidised glutathione.

Taken together, recent reports suggest that H_2_S is capable of inhibiting the production of ROS, scavenging and neutralising ROS and boosting the efficacy of endogenous anti-oxidant molecules (Figure 2). The net effect is protection of vascular function and future work is needed to further examine the potential therapeutic benefits of the anti-oxidant effects of H_2_S.

## Studies in vascular disease states showing vasculoprotective effects of H_2_S

### Hypertension

Hypotensive effects of H_2_S were first reported when administration of H_2_S donors *in vivo* to anaesthetised rats was found to induce a transient hypotensive effect [55]. The CSE-L-cysteine pathway is downregulated in spontaneously hypertensive rats and treating them with a H_2_S donor is protective, reducing blood pressure and vascular remodelling [56]. The most compelling evidence for the importance of H_2_S in blood pressure regulation is that mice deficient in CSE develop endothelial dysfunction and hypertension within 8 weeks of birth and that H_2_S replacement decreases systolic blood pressure in both CSE^−/−^ and CSE^+/−^ mice [8]. H_2_S is also reported to regulate plasma renin levels [57] and inhibit angiotensin converting enzyme (ACE) activity in endothelial cells [58]. Inhibitory effects on ACE could also contribute to the anti-remodelling effects, which involve H_2_S inhibition of collagen synthesis and smooth muscle proliferation in spontaneously hypertensive rats [59].

### Angiogenesis

H_2_S in implicated in the control of angiogenesis as NaHS treatment caused endothelial cell proliferation, adhesion, migration and tubule formation [60,61], with further work showing that vascular endothelial growth factor (VEGF) induced angiogenesis is mediated via H_2_S [61] and that H_2_S treatment *in vivo* increases collateral vessel growth, capillary density and blood flow in a hindlimb ischaemia model [62].

### Atherosclerosis

Atherosclerosis is a chronic immune-inflammatory, fibro-proliferative disease caused by lipid accumulation, affecting large and medium-sized arteries [63] Atherosclerosis is the most common underlying cause in the development of coronary artery disease. It has a multifactorial pathogenesis, involving vascular inflammation, recruitment and infiltration of monocytes, differentiation of monocytes to foam cells. This leads to increased reactive oxygen species generation resulting in an impairment of vascular endothelial function, by reducing NO bioavailability [64]. Further accumulation of foam cells and vascular smooth muscle cell proliferation lead to the formation of vascular lesions or plaques, which disrupt blood flow and reduce vessel compliance. A number of studies have indicated that H_2_S has many properties that may lead to the inhibition of atherogenesis (for review see [65]).

H_2_S donors have been shown to reduce inflammatory mediators, an effect that is dose-dependent and also influenced by delivery of H_2_S. Rapid delivery via NaHS is more likely to induce pro-inflammatory effects, whereas a more controlled delivery via the newer H_2_S donor GYY4137 produces mostly anti-inflammatory effects [66]. H_2_S treatment leads to decreased chemokine signalling [67] due to H_2_S-donor dependent downregulation of macrophage CX3CR1 receptor expression, and CX3CR1-mediated chemotaxis [67]. NaHS inhibited leukocyte adhesion in mesenteric venules, and importantly, inhibiting CSE enhanced leukocyte adherence and infiltration [68]. NaHS treatment reduced ICAM-1 levels in ApoE^−/−^ mice [69]. This adhesion molecule participates in adhesion strengthening, monocyte spreading and transendothelial migration thus contributes to the infiltration of inflammatory cells into the vessel wall [70].

Once leukocytes have traversed the vessel wall the next stage in atherogenesis is foam cell formation. H_2_S has been shown to inhibit hypochlorite induced atherogenic modification of purified LDL *in vitro*[71] and further studies have revealed that NaHS treatment inhibits macrophage expression of scavenger receptors (CD36 and scavenger receptor A) and acyl-coenzyme A:cholesterol acyltransferase-1, key proteins required for uptake of oxidized lipoproteins and subsequent cholesterol esterification required for foam cell production [72].

Administration of H_2_S donors lead to a number of effects on vessel remodelling. In one study, CSE expression was reduced, and endogenous H_2_S production decreased in blood vessels with balloon-injury induced neointima. The neointima formation was attenuated in animals treated with NaHS [73]. H_2_S is known to cause inhibition of proliferation [74], and induction of apoptosis [75] in human aortic vascular smooth muscle cells, and reduce collagen deposition [59]. CSE over-expression in human embryonic kidney cells inhibits proliferation [76] and importantly, a recent study showed that CSE-deficient mice have increased neointima formation, that was reversed with NaHS treatment [77].

**Figure 3:**
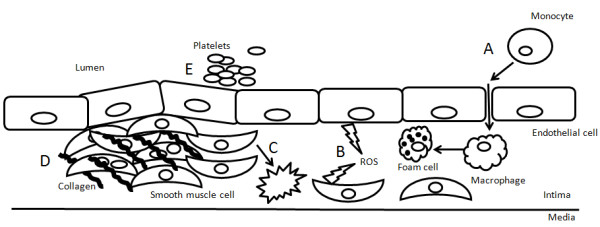
**Potential sites of vasculoprotective effects of H**_**2**_**S.** Cartoon depicting a cross section of the vascular wall showing the endothelium, intima containing smooth muscle cells overlaying the vascular media. **A**. H_2_S has been shown to decrease leukocyte adhesion and migration [60] and differentiation to foam cells [64]. **B**. H_2_S can inhibit the production of ROS [39,40] as well as scavenge ROS [35-38], protecting endothelial function. **C** H_2_S prevents proliferation [66] and promotes apoptosis of vascular smooth muscle cells [67]**D**. H_2_S prevents collagen deposition [51] and neo-intima formation [65]. **E** H_2_S can inhibit platelet adhesion [26] and aggregation [25].

NaHS treatment of ApoE^−/−^ mice on a high fat diet reduced atherosclerotic lesion area [69]. NaHS treatment has been shown to inhibit vascular smooth muscle cell calcification in both cell culture [78] and in a rat model of vascular calcification [79]. Additionally, NaHS treatment in fat fed ApoE^−/−^ mice improved endothelial function and reduced vascular oxidative stress. Plasma H_2_S levels are correlated with higher HDL and adiponectin levels and lower triglycerides and LDL/HDL ratio [80] in healthy human subjects, suggesting that increasing sulfide consumption may have cardiovascular benefits. Overall H_2_S has been shown to impede atherogenesis at all stages of the disease process (Figure 3). Taken together these effects all point towards an atheroprotective effect of endogenous H_2_S, that is elicited by endogenous H_2_S and that exogenous H_2_S application may be a useful therapeutic strategy to prevent vascular remodelling.

### Changes in expression of CSE in disease states

Altered expression of CSE and reduced endogenous H_2_S are observed in inflammation [68], atherosclerosis [69], diabetes [81], hypertension [56] and treatment with H_2_S donors has been repeatedly shown to be beneficial. The inverse relationship between plasma H_2_S levels and vascular disease strongly suggests a role for endogenous H_2_S in maintaining normal vascular functions.

## Conclusions

The field of H_2_S biology is new and exciting with regular reports of new developments in the literature. It is clearly an important mediator in the vascular system, contributing to vascular regulation and protection of cells from oxidative stress and the vascular injury that result from this and leads to vascular dysfunction. There is good evidence that H_2_S donor treatment has potential as a vasculoprotective agent for the prevention and reversal of cell damage that is implicit in many vascular disease states.

## Abbreviations

CBS: Cystathionine-β-synthase; CSE: Cystathionine-γ-lyase; MST: 3-mercaptopyruvate sulfurtransferase; PGI2: Prostacyclin; ET-1: Endothelin-1; AII: Angiotensin II; EDHF: Endothelium-derived hyperpolarising factor; NADPH: Nicotinamide adenine dinucleotide phosphate; Nox: NADPH oxidase; ROS: Reactive oxygen species; SOD: Superoxide dismutase; CAT: Catalase; MPO: Myeloperoxidase; GPx: Glutathione peroxidase; GSH: Reduced glutathione; GSSG: Oxidized glutathione; ACE: Angiotensin converting enzyme; VEGF: Vascular endothelial growth factor; LDL: Low density lipoprotein; HDL: High density lipoprotein.

## Competing interests

The authors declare that they have no competing interests.

## Authors’ contributions

JH, HN and ES wrote the manuscript. All authors have read and approved the final manuscript.
